# Determining the Impact of Medical Arabic Workshops on Medical Students' Preparedness and Communication Skills With Arabic-Speaking Patients

**DOI:** 10.7759/cureus.87685

**Published:** 2025-07-10

**Authors:** Oase Sbei, Yasser Almansour, Belal Sbei, Yusra Sannah, Mukhlis Alabdelrazzak, Haytham Alqasmi, Omar Abdalla, Alexandra Turfe, Anas Kutait

**Affiliations:** 1 Education, Wayne State University School of Medicine, Detroit, USA; 2 Medicine, Michigan State University College of Human Medicine, East Lansing, USA; 3 Research, University of Michigan, Ann Arbor, USA; 4 Medicine, Wayne State University School of Medicine, Detroit, USA; 5 Gastroenterology and Hepatology, Henry Ford Health System, Detroit, USA

**Keywords:** arabic and english language, curriculum assessment, medical arabic, medical education curriculum, workshop training

## Abstract

Introduction

While Wayne State University School of Medicine (WSUSOM) serves a large Arab-American patient population, medical students have limited opportunities for formal instruction in medical Arabic, which may hinder effective communication with Arabic-speaking patients. To address this gap, a series of medical Arabic workshops was implemented, focusing on medical terminology and patient interaction. This study evaluates the impact of these workshops on students' self-reported preparedness and confidence when interacting with Arabic-speaking patients. Notably, there is a lack of prior US-based studies examining the effectiveness of structured medical Arabic training for medical students, further underscoring the novelty and relevance of this research.

Methods

An anonymous survey was distributed to WSUSOM students who attended one or more medical Arabic workshops. The survey utilized a five-point Likert scale (1 = never, 5 = always) to assess domains such as communication confidence, comprehension preparedness, and perceived training utility. A total of 119 students completed the pre-workshop survey, and 110 completed the post-workshop survey. Unpaired t-tests were used to compare weighted averages across domains before and after the workshops, with p-values <0.05 considered significant.

Results

Participation in the medical Arabic workshops was associated with statistically significant improvements in students’ confidence and preparedness when interacting with Arabic-speaking patients. Pre-workshop, the weighted average score for confidence in understanding Arabic in clinical settings was 2.18 (SD = 1.34), which increased to 3.14 (SD = 1.15) post-workshop (p < 0.0001). Similarly, students’ confidence in communicating health information rose from an average of 2.10 (SD = 1.21) to 3.17 (SD = 1.09) after attending the workshops (p < 0.0001). Additionally, the proportion of students reporting regular use of Arabic in clinical encounters remained high, but post-workshop responses indicated a reduction in the frequency with which students needed clarification during patient interactions. Overall, the majority of participants agreed that the workshops enhanced their clinical skills and preparedness to serve Arabic-speaking patients.

Conclusions

Medical Arabic workshops effectively improved medical students’ confidence and readiness to engage with Arabic-speaking patients. These findings suggest that targeted language and cultural competency training may enhance communication and clinical performance, and that integration of similar programs into the formal medical school curriculum could help address language barriers in patient care.

## Introduction

Barriers to healthcare for Arabic-speaking patients with limited English proficiency (LEP) have been documented extensively, highlighting the significant role of language and cultural differences in shaping access to and quality of care. Arabic-speaking immigrants with LEP are more likely to report poorer self-rated health than both English-speaking immigrants and US-born Arab Americans, suggesting that language preference is a key factor influencing health perceptions and service utilization [1]. In a study conducted in Iowa, nearly half of Arabic-speaking participants reported facing one or more barriers, such as lack of health insurance, high co-payments, and language difficulties, that hindered their ability to access preventive healthcare services. Notably, those without health insurance were far less likely to seek regular physician visits, even when living with chronic illnesses [2]. Additionally, Arabic-speaking students demonstrated significantly lower comprehension and performance on English-language health questionnaires compared to their Arabic versions, indicating that language barriers directly affect understanding and engagement with healthcare information [3].

A key challenge faced by LEP patients is language discordance, in which patients and their healthcare providers do not share equivalent proficiency in the same language [4]. Unlike a language barrier, which suggests an insurmountable communication issue, language discordance refers to difficulties in achieving full understanding. Even when some overlap of common language is shared, differences in fluency, dialect, or medical terminology can still lead to miscommunication. Similar to language barriers, physician-patient language discordance is linked to poor health outcomes, while language concordance has been shown to improve outcomes and satisfaction of patients with LEP [5,6]. More specifically, language concordance in clinical encounters has been associated with increased patient follow-up, avoidance of medication complications, and reduced use of emergency care services when compared to language discordant encounters [6].

One avenue for addressing language discordance is through second-language communication skills training in medical schools. Training in English alone has been shown to leave medical students unconfident with patient communication in their native language [7]. This trend is observed in medical schools globally. Given that English is the leading language of medical education around the world [8], several studies highlight the challenges of English-only patient-communication training for medical students in various countries, including the United Arab Emirates, Saudi Arabia, and South Africa. In addition to low self-reported confidence, medical students in these areas reported difficulties in communicating, empathizing, and/or eliciting patient emotions in their native language [7,9-11].

The American Medical Association (AMA) strongly advocates for the inclusion of language training in medical education to enhance communication with LEP patients. Recognizing the critical role of effective communication in patient care, the AMA encourages all Liaison Committee on Medical Education (LCME)- and American Osteopathic Association (AOA)-accredited US medical schools to offer medical second language courses as electives. While some US medical schools have welcomed student-led medical Arabic initiatives, there remains a lack of integration of formal second-language programming in medical school curricula. This may be, in part, due to a limited understanding of the impact of language training in medical Arabic on the preparation of medical students for interactions with Arabic-speaking patients in the US.

We believe that physicians in training must be equipped to communicate effectively not only with patients from diverse backgrounds but especially with regionally prevalent minority populations. According to the Pew Research Center’s analysis of data from the US Census Bureau and the 2021 American Community Surveys, the greater Detroit region has the highest number of Arabic speakers among all US metropolitan areas [12]. Approximately 190,000 Arabic speakers reside in the Detroit-Warren-Dearborn metro area, accounting for 13% of the estimated 1.45 million Arabic speakers in the United States [12]. Moreover, the area holds the highest concentration of Arabic speakers, with 205,164 individuals (4.7% of the population aged five and older) speaking Arabic at home [12]. Of these, only 127,201 (62%) are proficient in English, leaving approximately 77,962 (38%) with LEP [12]. Nationally, Arabic is the fastest-growing language among the top 10 non-English languages spoken in the US [13]. Estimates suggest that 457,271 out of over 1.3 million Arabic speakers in the US speak English less than “very well” [14], placing them at increased risk of experiencing language barriers in healthcare. Despite these demographics, many graduating medical students lack training in second or heritage languages. Evaluating medical student perspectives may provide valuable insight into the unmet need for medical Arabic communication training in US medical education programs.

No prior studies, to our knowledge, assess the impact of medical Arabic programs on Arabic-proficient students attending US medical schools, although some programs have been implemented overseas. Medical Arabic programming, including simulated patient interviews, enhanced students' communication skills and confidence with Arabic-speaking patients [7,15,16]. Yet, questions remain regarding the self-reported utility and demand for medical Arabic communication training for US medical students with Arabic proficiency, especially those in areas with large Arabic-speaking populations.

In recent years, medical students at Wayne State University School of Medicine (WSUSOM) developed an extracurricular series of medical Arabic workshops to better understand and address this need. This study aims to evaluate the impact of medical Arabic workshops at WSUSOM on the preparedness and confidence of medical students in communicating with Arabic-speaking patients.

## Materials and methods

The study was designed as a cross-sectional, survey-based evaluation conducted at Wayne State University School of Medicine (WSUSOM) in Detroit, Michigan, between May 2024 and November 2024. It aimed to assess the impact of an extracurricular Medical Arabic Workshop Series on medical students’ self-reported confidence and preparedness when communicating with Arabic-speaking patients. This project was classified as non-human subjects research under the Common Rule at 45 CFR 46 and FDA regulations and therefore did not require institutional review board approval.

The Medical Arabic Workshop Series consisted of six sessions held over a six-month period. Each session lasted approximately 90 minutes and was facilitated by Arabic-speaking medical students who had prior training in clinical communication skills. Workshop sessions incorporated multiple teaching methods, including didactic instruction of clinical vocabulary, peer-to-peer role-play exercises, small-group discussions, and distribution of educational handouts summarizing medical Arabic phrases and terms. Content focused on essential clinical encounters such as patient intake, symptom assessment, medication counseling, discharge instructions, and culturally sensitive communication practices. Workshops were offered as a voluntary, service-learning opportunity that students could use to fulfill WSUSOM’s required service-learning hour requirement.

Students who attended one or more sessions were eligible to participate in the study. Participants were recruited through email notifications, workshop announcements, and flyers posted around WSUSOM. An anonymous, electronic survey was distributed using Google Forms (Google, Mountain View, CA). Participation was voluntary, and students could opt out at any time. No personally identifiable information was collected, and responses were unlinked across timepoints to protect anonymity.

Survey instruments were developed specifically for this study, drawing on prior literature related to language concordance and communication confidence. Questions were reviewed by clinical faculty advisors for face validity, and the instrument was piloted among a small group of medical students to ensure clarity and relevance. However, no formal psychometric validation was performed.

Students completed a pre-workshop survey at the start of their first session and a post-workshop survey at the conclusion of their final session or once the series ended. The pre-workshop survey included three quantitative questions, while the post-workshop survey contained four quantitative questions, all using a five-point Likert scale (1 = never to 5 = always). These questions assessed self-reported domains including communication preparedness, confidence in comprehension, ability to educate Arabic-speaking patients, frequency of needing clarification during interactions, and Arabic language use in clinical settings. An optional open-ended question was included for qualitative feedback; however, these responses were not systematically analyzed.

Although the survey was anonymous, the pre-workshop instrument captured information that served as a proxy for baseline Arabic proficiency by assessing students’ comfort and frequency of use in clinical scenarios. Notably, none of the participants identified Arabic as their first language, ensuring the study population reflected learners with limited prior fluency.

A total of 119 students completed the pre-workshop survey, and 110 students completed the post-workshop survey, with differences due to early departures, scheduling conflicts, or incomplete responses. Data was collected through Google Forms and securely stored, accessible only to principal investigators. All quantitative data were exported to Microsoft Excel (Microsoft Corporation, Redmond, WA) for statistical analysis. Descriptive statistics, including means, standard deviations, and standard errors of the mean, were calculated for each survey domain. To evaluate changes between pre- and post-workshop responses, unpaired two-tailed t-tests were used, given the anonymity of the data and lack of matched pairs. A p-value of less than 0.05 was considered statistically significant. Changes in weighted averages were visualized using bar graphs generated in Microsoft Excel, and Likert response distributions were illustrated to show shifts in self-reported confidence and preparedness. No subgroup analyses based on baseline proficiency or number of sessions attended were performed due to the anonymous nature of survey collection.

## Results

Preliminary analysis revealed a statistically significant improvement in students’ confidence and communication skills when interacting with Arabic-speaking patients following participation in the Medical Arabic workshops (p < 0.0001 for both metrics). The majority of students reported that the sessions improved their preparedness and ability to engage with patients more effectively.

**Figure 1:**
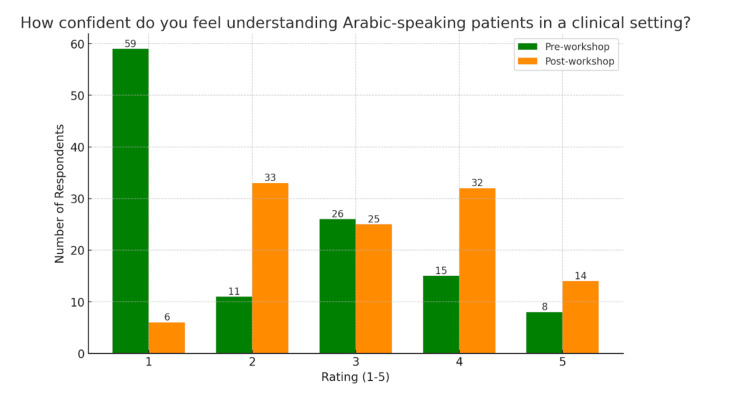
Comprehension confidence ratings represents pre- and post-workshop. Medical students rated their confidence regarding their ability to understand Arabic in the clinical setting on a scale of 1-5. The pre-workshop weighted average rating is 2.18 with a standard deviation of 1.34 (n = 119). The post-workshop weighted average rating is 3.14 with a standard deviation of 1.15 (n = 110).

**Figure 2:**
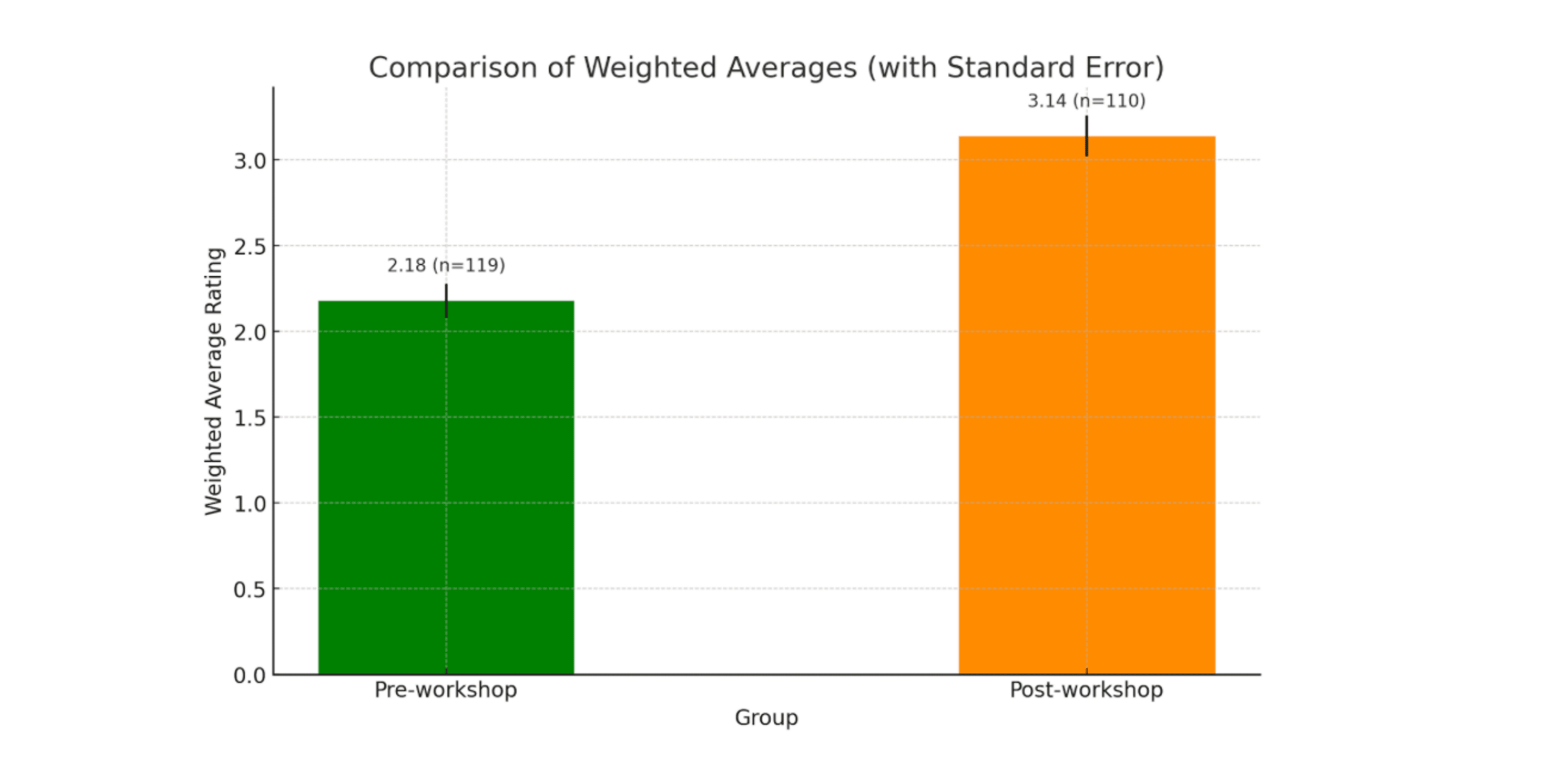
Weighted averages of comprehension confidence ratings. The figure represents the pre- and post-workshop weighted averages (with standard error bars) regarding students' ability to understand Arabic-speaking patients in the clinical setting on a scale of 1-5, with a mean difference of 0.96 and an unpaired t-test yielding a t = 5.7955 (p < 0.0001).

The pre-survey data showed that 97 (81.5%) students rated their confidence in understanding Arabic in the clinical setting as 3 or lower on a five-point scale, with a weighted average of 2.18 (SD = 1.34, n = 119). As shown in Figure 1, the distribution was heavily skewed toward low confidence levels, underscoring a clear gap in students' self-assessed preparedness. In contrast, Figure 2 shows that the post-workshop average rose to 3.14 (SD = 1.15, n = 110), with responses more evenly distributed across the scale. This change represents a mean difference of 0.96, supported by an unpaired t-test result of t = 5.80 (p < 0.0001), indicating statistically significant improvement in comprehension confidence.

**Figure 3:**
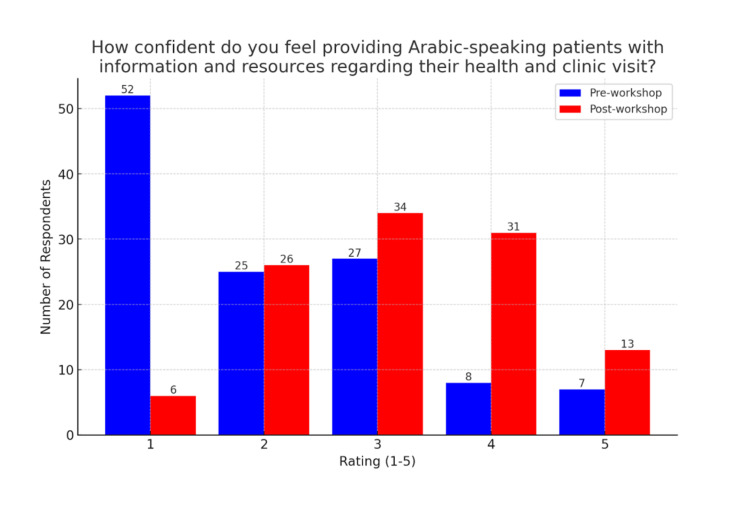
Communication confidence ratings pre- and post-workshop. The figure represents pre- and post-workshop survey results regarding students' confidence in providing Arabic-speaking patients with information and resources regarding their health and clinic visits on a scale of 1-5. The pre-workshop weighted average rating is 2.10 with a standard deviation of 1.21 (n = 119). The post-workshop weighted average rating is 3.17 with a standard deviation of 1.09 (n = 110).

**Figure 4:**
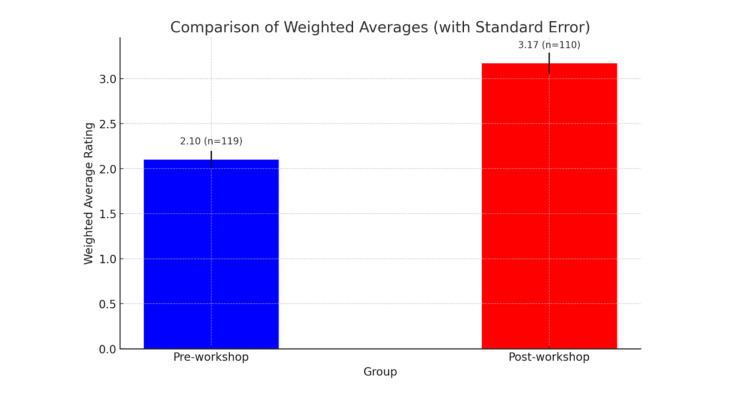
Weighted averages of communication confidence ratings. The figure represents the pre- and post-workshop weighted averages (with standard error bars) regarding students' confidence in providing Arabic-speaking patients with information and resources regarding their health and clinic visits on a scale of 1-5, with a mean difference of 1.07 and an unpaired t-test yielding a t = 7.0106 (p < 0.0001).

Similarly, students’ confidence in communicating health information to Arabic-speaking patients improved notably. Pre-workshop, the average self-rating was 2.10 (SD = 1.21, n = 119), while post-workshop ratings increased to 3.17 (SD = 1.09, n = 110), as illustrated in Figures 3, 4. This mean difference of 1.07 was also statistically significant (t = 7.01, p < 0.0001), further affirming the positive impact of the workshop.

**Figure 5:**
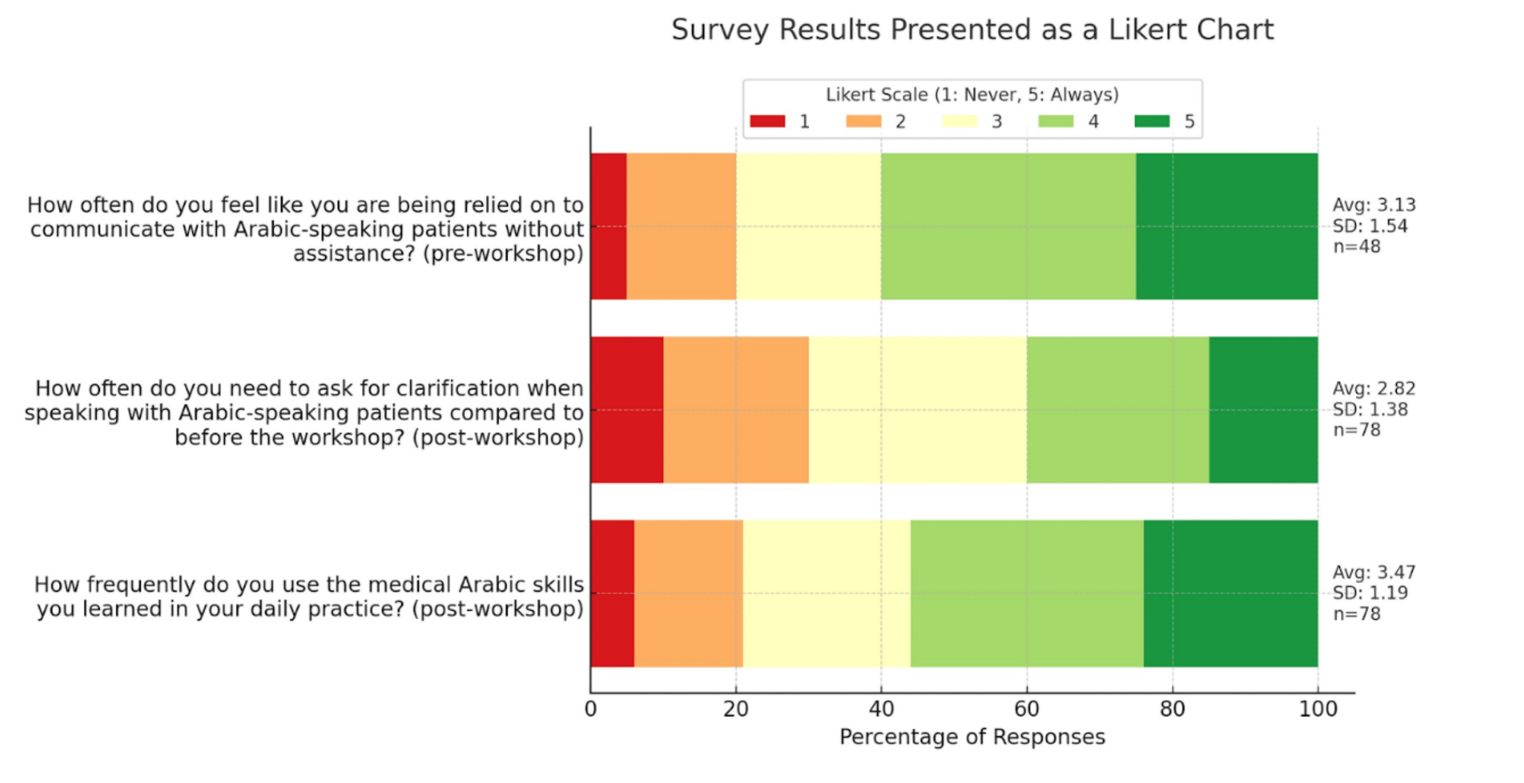
Medical students' experiences in communicating in Arabic in clinical encounters. The figure displays percentages of responses (1-5) to questions that were isolated to either pre- or post-workshop surveys. Results show that 71 (60%) students felt that they were either always or almost always relied on to communicate with Arabic-speaking patients without assistance. Of respondents, 71 (60%) felt that at most they sometimes had to ask for clarification when speaking to Arabic-speaking patients as compared to before the workshop. Only about 24 (20%) students reported never or almost never using medical Arabic skills in their daily practice.

Figure 5 provides insight into students' practical experiences with Arabic in clinical settings. Prior to the workshops, 71 (60%) students reported that they were always or almost always relied upon to communicate with Arabic-speaking patients without assistance, underscoring the demand for Arabic language use in clinical encounters. In a separate post-workshop question, 71 (60%) students indicated that they only sometimes or less frequently needed clarification when speaking with Arabic-speaking patients. Additionally, following the workshop, only 24 (20%) students reported never or almost never using Arabic in their medical practice, suggesting that the majority continue to encounter situations where Arabic language skills are applied.

## Discussion

This study aimed to evaluate the impact of medical Arabic workshops on the preparedness and communication skills of medical students at WSUSOM when interacting with Arabic-speaking patients. The results demonstrated a statistically significant improvement in students’ self-reported comprehension and communication confidence following the workshops. Given the large Arabic-speaking population in the Detroit metropolitan area and the broader challenges faced by LEP patients, these findings highlight the potential benefits of integrating medical Arabic training into medical education.

The findings of this study align with broader research indicating that language concordance improves healthcare experiences and outcomes for LEP patients. Prior studies have demonstrated that when patients and healthcare providers share a common language, there is increased adherence to medical advice, a reduction in emergency care reliance, and an overall improvement in patient satisfaction [5,6]. These benefits stem from improved trust between providers and patients, as well as a more accurate exchange of medical information, leading to better diagnosis and treatment outcomes. This study contributes to these findings by showcasing that even short-term language training programs can help bridge communication gaps and better prepare medical students for interactions with linguistically diverse patient populations.

Research has also highlighted the importance of structured language training in medical education. Programs that incorporate targeted language instruction, along with exposure to clinical scenarios, have been found to enhance students’ confidence and competence in communicating with patients who have LEP [15,16]. Such programs have demonstrated that hands-on practice, simulated patient interactions, and peer-assisted learning strategies significantly improve students’ ability to navigate real-world clinical encounters. This study builds on that understanding by demonstrating that structured training, even when provided in an extracurricular format, can have a measurable impact on student preparedness and confidence levels in clinical settings.

Furthermore, language barriers in healthcare have been well-documented as a major factor contributing to health disparities. Patients who do not speak the primary language of their healthcare provider often face miscommunication, decreased trust, and lower rates of healthcare utilization [1,2]. Studies have consistently shown that linguistic challenges contribute to poorer health outcomes, including delayed diagnoses, improper medication usage, and increased rates of hospitalization. The results of this study reinforce the argument that language training should be considered a key component of medical education, as it plays a role in improving healthcare accessibility and reducing disparities among linguistically diverse populations.

In addition, previous research has pointed out that even when medical students are fluent in a secondary language, they often lack confidence in using medical terminology and effectively conducting patient interviews in that language [7]. Language training programs specifically tailored to medical contexts have been found to alleviate these concerns by providing structured exposure to medical conversations, increasing students’ ability to communicate effectively and comfortably in multilingual clinical settings. This study extends that body of knowledge by highlighting the direct benefits of short-term language workshops in fostering such confidence.

Beyond immediate improvements in confidence, medical language training may yield long-term benefits for both patient outcomes and physician practice patterns. Students who gain early exposure to language-concordant care may be more likely to pursue practice in underserved communities, increase their cultural humility, and develop sustained confidence in cross-cultural encounters. Over time, such training could contribute to the development of a more linguistically and culturally responsive physician workforce. Integrating language skills into the formal medical curriculum, especially for populations with significant local presence, such as Arabic speakers in metro Detroit, can strengthen patient-provider relationships, promote continuity of care, and reduce structural barriers in healthcare delivery. Future longitudinal studies are needed to evaluate whether early language interventions translate into long-term practice behavior, patient satisfaction, and clinical outcomes.

One of the key strengths of this study is its quantitative approach, utilizing pre- and post-workshop surveys with statistical analysis to assess the effectiveness of the workshops. The large sample size (n = 119, pre-workshop; n = 110, post-workshop) adds robustness to the findings. Additionally, this study is among the first to systematically evaluate the impact of medical Arabic training on US medical students, contributing to a growing body of literature advocating for enhanced linguistic training in medical curricula.

Despite its strengths, this study has several limitations. First, it relied on self-reported data, which may be subject to response and recall bias. Future research should incorporate objective assessments, such as standardized patient encounters or faculty evaluations, to more accurately measure students’ proficiency. Second, the study did not collect data on participants’ baseline Arabic language proficiency, which may have influenced their perceived improvement. Future studies should assess initial language competency to better stratify and interpret outcomes.

Another limitation is the absence of patient perspectives. Evaluating patients’ understanding and satisfaction with student communication before and after the workshops would provide a more comprehensive assessment of the program’s effectiveness and its impact on clinical care. Additionally, the workshop format primarily consisted of lecture-based instruction, with limited opportunities for small-group learning or peer-assisted practice, approaches shown to enhance language acquisition and retention. Future iterations should incorporate role-playing, case-based discussions, and direct patient interaction to reinforce practical skills.

Given the positive outcomes observed, further research should explore the longitudinal impact of medical Arabic training on graduates’ clinical performance and patient outcomes beyond residency. Expanding this model across multiple medical institutions and diverse student populations would improve the generalizability of findings. Moreover, integrating a qualitative component, such as in-depth interviews with both students and Arabic-speaking patients, could yield valuable insights into the real-world benefits and challenges of language concordance in clinical settings.

Lastly, the success of this program supports the expansion of similar workshops for other commonly spoken minority languages, such as Spanish, Mandarin, and Vietnamese. Such initiatives may play a critical role in improving communication, reducing health disparities, and advancing equitable care for LEP populations across the healthcare system.

The findings of this study emphasize the importance of integrating medical Arabic training into medical curricula to better equip future physicians for effective communication with Arabic-speaking patients. As the Arabic-speaking population in the US continues to grow, medical schools should consider implementing structured language programs to enhance healthcare accessibility and equity. The results support the need for institutional recognition of language concordance as a crucial factor in patient-centered care, aligning with broader public health initiatives aimed at reducing health disparities for LEP populations.

## Conclusions

The medical Arabic workshops appear to play a crucial role in improving medical students' abilities to communicate effectively with Arab-speaking patients. The findings suggest that such workshops should be integrated more formally into the medical curriculum to better serve the diverse patient population in the Midwest. These findings highlight the positive impact of medical Arabic training workshops in regions like the Midwest, especially in Southeast Michigan, where a large population of Arabic-only speaking patients reside. Integrating this education more formally into the curriculum would better prepare healthcare providers for working with diverse patient populations in these areas, helping to overcome language barriers and improve access to care for Arabic-speaking patients.

## Appendices

Survey questions

Pre-survey: https://forms.gle/qcgEomQm4MCdXP2u8

NAAMA (National Arab American Medical Association) NextGen Medical Arabic Workshop 

Thank you for your interest in our Medical Arabic Sessions!

Please fill out the survey based on your experience with our Medical Arabic series.

1. How confident do you feel in understanding Arab-speaking patients in a clinical setting?

*

Least confident

1

2

3

4

5

Most confident

2. How confident do you feel in providing Arab-speaking patients with information and resources regarding their health and clinic visit?

*

Least confident

1

2

3

4

5

Most confident

3. How often do you feel like you are being relied on to communicate with Arab-speaking patients without any assistance?

Always

1

2

3

4

5

Never

4. What do you hope to get out of our medical Arabic sessions?

Post survey: https://forms.gle/dQP3DD26cRksuHEc9

NAAMA NextGen Medical Arabic Workshop 

Thank you for attending!

Please fill out the survey based on your experience with our Medical Arabic series.

1. How confident do you feel in understanding Arab-speaking patients in a clinical setting?

*

Least confident

1

2

3

4

5

Most confident

2. How often do you need to ask for clarification when speaking with Arabic-speaking patients compared to before the workshop?

*

Always

1

2

3

4

5

Never

3. How frequently do you use the medical Arabic skills you learned in your daily practice?

*

Always

1

2

3

4

5

Never

4. How confident do you feel in providing Arab-speaking patients with information and resources regarding their health and clinic visit?

*

Least confident

1

2

3

4

5

Most confident

Please provide any feedback you wish to share regarding the event or your experience.
